# Influence of exoskeleton stiffness on primary afferent feedback during stretch perturbations of isolated muscle-tendon unit

**DOI:** 10.1101/2025.11.03.686366

**Published:** 2025-11-05

**Authors:** Amro A. Alshareef, Paul Nardelli, Surabhi N. Simha, Timothy C. Cope, Lena H. Ting, Gregory S. Sawicki

**Affiliations:** 1 Woodruff School of Mechanical Engineering, Georgia Institute of Technology, Atlanta, Georgia, USA; 2 School of Biological Sciences, Georgia Institute of Technology, Atlanta, Georgia, USA; 3 Coulter Department of Biomedical Engineering, Emory University and Georgia Institute of Technology, Atlanta, Georgia, USA; 4 Department of Rehabilitation Medicine, Emory University, Atlanta, Georgia, USA

**Keywords:** sensory feedback, exoskeletons, proprioception

## Abstract

Exoskeletons assist and augment human movement, but their effects on proprioceptive feedback remain poorly understood. We examined how parallel exoskeleton stiffness influences primary muscle spindle firing. In an anesthetized rat preparation, controlled stretches of the medial gastrocnemius were applied with springs (0–0.5 N/mm) attached in parallel to the muscle-tendon unit (MTU) to simulate passive exoskeleton assistance. Fascicle length was measured with sonomicrometry, force and MTU length with a servo motor, and spindle instantaneous firing rate (IFR) with dorsal root recordings. Increasing exoskeleton stiffness decreased biological muscle force (3.1 ± 0.6 N to 1.6 ± 0.6 N, p < 0.001) and stiffness (4.4 ± 1.5 N/mm to 2.3 ± 1.3 N/mm, p < 0.01), while fascicle length increased (7.9 ± 1.3 mm to 8.3 ± 1.5 mm, p < 0.005). Despite these altered mechanics, spindle firing did not significantly change, and showed weak correlations with muscle length, velocity, force, and yank (R^2^ ≤ 0.14). These results indicate that exoskeleton stiffness modifies fascicle dynamics without altering spindle firing. Previously proposed models of primary afferent firing did not sufficiently explain these results. This is the first in situ investigation of exoskeleton effects on primary afferent feedback during active contractions.

## Introduction

Exoskeletons and exo-suits are emerging as a viable technology for restoring, assisting or augmenting human movement. In the past decade, numerous exoskeletons have been developed that have been shown to reduce the metabolic effort of the user in walking and running tasks (1–4), increasing joint power (5), reducing muscle activity (6), and assisting with training and rehabilitation (7). Recently, exoskeleton research has shifted toward enhancing performance during unsteady locomotion tasks that require dynamic force response - like maneuvering to change direction or stabilizing to recover balance (8–13). Indeed, powered ankle exoskeletons can enhance standing balance recovery by acting faster than natural human response times while also reducing the effort required to stabilize in response to body pitch perturbations (8, 9). In this case the exoskeleton was ‘all knowing’ and could act instantaneously, but the real physiological system involves a closed sensorimotor loop. Despite the continually expanding development of exoskeleton controllers for these applications, little focus has been placed on how these exoskeleton assistance profiles influence the user’s internal sensorimotor control loop. While the exoskeleton can enhance motor output, it may also have undesirable impacts on sensory signaling as an external system interacting with the human (8, 14). Here, we aimed to isolate the effects of exoskeleton assistance on proprioceptive feedback during controlled, active muscle dynamics that simulate human muscle dynamics during balance recovery responses.

Proprioceptive feedback has a large influence on motor control and stability. Muscle spindle sensory organs provide one such source of feedback, lying within the muscle belly and form a sophisticated feedback control loop that influences motor control (15). Muscle spindle feedback has significant impact on inter-joint coordination, muscle stiffness regulation, and movement and posture control and dynamics in human and animal models (16–22). Furthermore, certain indicators used to study stability and balance in humans, including within-step modulation of muscle activity, swing leg step placement during perturbations, and accurate postural and joint position control, depend on spindle feedback (23–25). While there is a heavy influence of sensory feedback from other avenues that are often studied in clinical applications, including visual and vestibular feedback, this previous work indicates a critical role that muscle sensory organ feedback plays in how the neuromuscular system controls movement that is independent of other sensory feedback loops. Most work quantifying this proprioceptive feedback has been limited to animal models, due to the ability to safely and effectively administer chemical agents, invasively measure afferent firing rates, isolate motor units, and highly control muscle tendon unit dynamics (19, 26, 27). Experiments investigating sensory feedback with exoskeletons have been done in humans but have used indirect measures of sensorimotor control such as metrics of stability and agility or fascicle dynamics (13, 14, 28). Investigations with direct primary afferent feedback measurements, on the other hand, have largely investigated passive muscle stretches and limited active stretch paradigms without exoskeleton influence (17, 26, 29–31).

In part due to the limitations above, a robust model that can accurately predict the relationship between muscle afferent feedback and contractile dynamics in various contexts has not been developed. Classically, muscle spindle feedback is believed to be influenced by muscle tendon unit (MTU) level kinematics. Due to the connective tissues in parallel and series to the intra/extrafusal muscle fibers, however, whole MTU kinematics likely do not well-represent the force or length changes experienced by spindles (26, 32). Furthermore, afferent firing behavior is heavily dependent on the mechanical context in which the muscle is stretched and loaded, and whether the muscle is active (17, 26, 29–32). Contractile force and yank acting on the intrafusal fibers, for example, has been proposed as a more accurate predictor of spindle instantaneous firing rate (IFR) during passive MTU stretches (26, 33). Regardless of the mechanism underlying spindle feedback, the altered muscle dynamics caused by added exoskeleton assistance likely also have an effect. During both cyclic and balance recovery tasks, exoskeletons act mechanically in parallel to a joint, either passively through a spring in parallel with the musculoskeletal tissues, or actively through a motor coupled to the joint, they introduce an external influence on the dynamics of the MTU (34). By providing an assistive torque, the biological force required by the muscle decreases and the fascicle length excursion increases, decreasing the biological muscle stiffness (34–36). These effects in turn influence muscle activation dynamics and the point along the muscle’s force-length and force-velocity curves at which it operates (11). While the exoskeleton assistance can enhance overall motor output, these effects on the internal muscle dynamics may cause unintended impacts on the proprioceptive feedback loops that are also important for accomplishing these tasks. A novelty of using an exoskeleton in parallel with the MTU to investigate sensory feedback is that the exoskeleton decouples the direction of kinematic and kinetic changes experienced by the muscle during eccentric contractions, where the muscle is generating force while lengthening. Not only do these contractions allow a clearer investigation of the independent effects of muscle kinematics and kinetics on primary afferent firing, but they are also highly relevant and understudied behavioral tasks.

Here we demonstrate how muscle spindle firing, reported as Instantaneous Firing Rate (IFR), is influenced by increased exoskeleton stiffness during eccentric contractions in an acute rat preparation. We first developed a simple mechanical model (representing active and passive elements of the MTU) consisting of series (representative of aging or tendinosis) (37) and parallel springs (representing passive exoskeletal assistance) to generate predictions across a range of exoskeleton stiffnesses. We used these results to inform our in-situ tests, in which we recreated the muscle dynamics observed during standing perturbation paradigms in humans with exoskeletons by imparting ramp stretches to the MTU with a spring in parallel (8). This is the first in-situ investigation of the effects of exoskeleton assistance on spindle feedback in an animal model during active contractions. We predicted that IFR would increase alongside the muscle fascicles’ length and velocity changes, resulting in an increase in IFR as parallel exoskeleton stiffness increased.

## Results

In this experiment, we successfully recreated the muscle dynamics observed in human standing balance experiments with exoskeleton assistance, where the biological moment and exoskeleton torque trade off across exoskeleton stiffness conditions. We observed a significant decrease in contractile muscle force and an increase in fascicle length change as exoskeleton stiffness increased. The fascicle length increased from 7.89 ± 1.29 mm to 8.33 ± 1.45 mm (p < .005) and the fascicle force decreased from 3.13 ± 0.64 N to 1.59 ± 0.56 N as parallel stiffness increased from 0 N/mm to 0.5 N/mm (p < .001) (Fig. 3B). Figure 3A shows the fascicle length, muscle force, and IFR from a representative animal and cell across all exoskeleton stiffness conditions, with the yellow stars on the x-axis indicating location of muscle stimulation.

Overall, adding the parallel spring did not significantly alter fascicle displacement relative to MTU displacement during stretch (Fig. 2D). The intended trade-off between muscle stiffness and exoskeleton stiffness was achieved, indicating that the experimental set up properly recreates the muscle dynamics observed in humans when wearing a passive exoskeleton (Fig. 2C) (34). The measured muscle stiffness and total MTU stiffness respectively decreased from 4.38 ± 1.46 N/mm and 1.08 ± 0.38 N/mm to 2.29 ± 1.32 N/mm and 0.61 ± 0.33 N/mm as exoskeleton stiffness increased from 0 N/mm to 0.5 N/mm (p < .01) (Fig. 2D). The RFD did not change significantly across exoskeleton conditions.

While the in-situ measured IFR dynamic response with no significant change across the range of exoskeleton stiffness (p > .05), the spike count (number of spikes on the rising phase of the ramp stretch) significantly increased from 17.12 ± 7.02 to 23.03 ± 6.41 as parallel stiffness increased from 0 N/mm to 0.5 N/mm (p < .05) (Fig. 3B). This is likely because in the higher activation conditions, the muscle initially before stretching. During this initial shortening phase, the spindle fibers are relaxed and then engage in a more rapid lengthening during the subsequent stretch, resulting in fewer overall spikes during the ramp, but reaching the same dynamic response as the passive condition with no initial shortening.

IFR was generally poorly correlated with muscle dynamics, including muscle length (R2 = 0.1345, p<.0001), muscle velocity (R2 = 0.14, p<.0001), muscle force (R2 = 0.0769, p<.0001), and muscle yank (R2 = 0.0015, p>.05), during the controlled portion of the ramp stretch. IFR also poorly correlated to other proposed IFR model variables (30), including muscle power (R2 = 0.1241, p<.0001) and muscle velocity raised to the power of 0.6 (R2 = 0.1414, p<.0001) (Fig. 3C).

## Discussion

**Figure 1.**
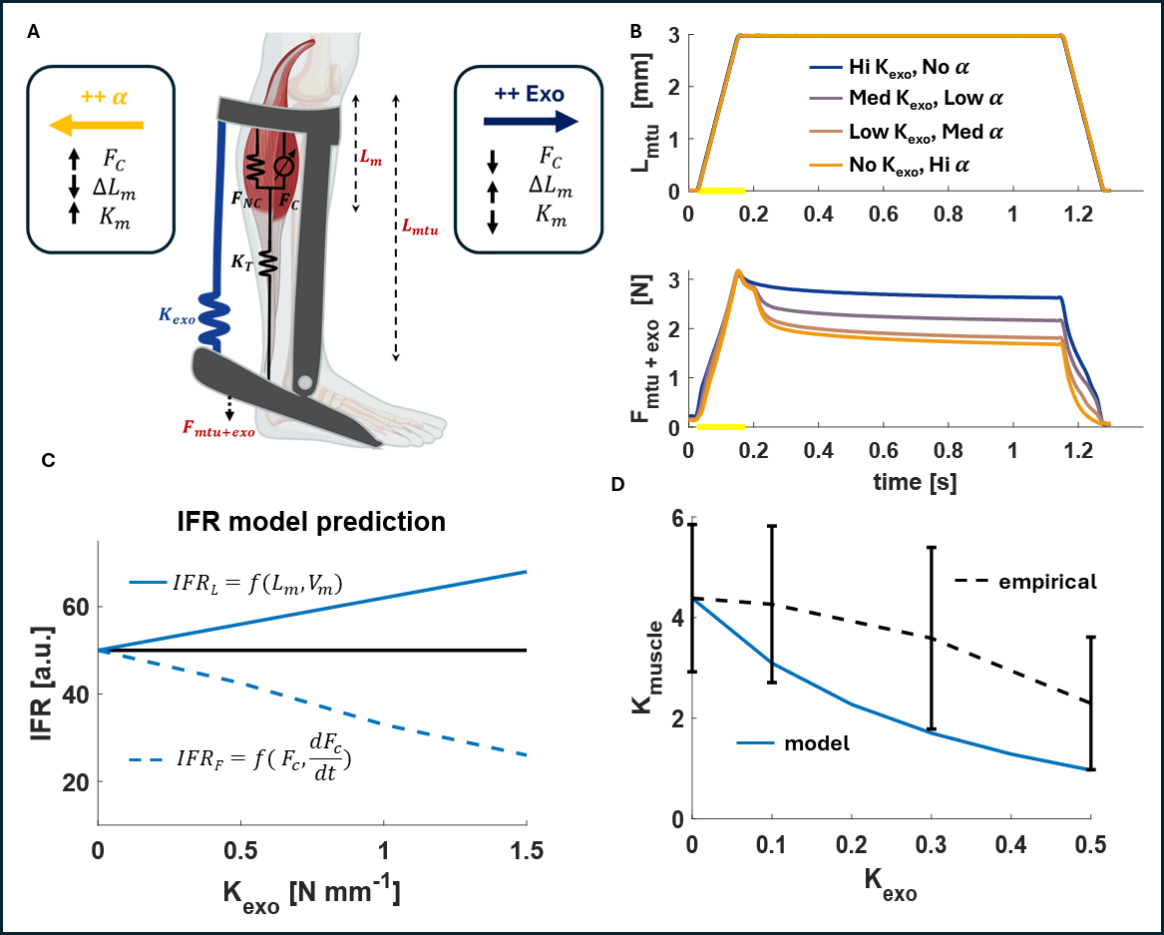
Simple muscle model development and IFR prediction. A) a simple hill-type muscle model with contractile and non-contractile muscle forces contributing to the biological muscle force, the tendon as a series elastic element, and the addition of the exoskeleton as a parallel elastic element to the whole MTU. With a constant total system stiffness and length change (B) the total force and length change of the MTU+Exoskeleton system was held constant across all conditions by trading off between muscle activation and exoskeleton stiffness contribution to the total force. C) The resulting predictions based on kinetic and kinematic based IFR model predictions. D) Model prediction of muscle stiffness versus exoskeleton stiffness based on equation 4 compared to empirical observation in this study. Error bars are SEM.

**Figure 2.**
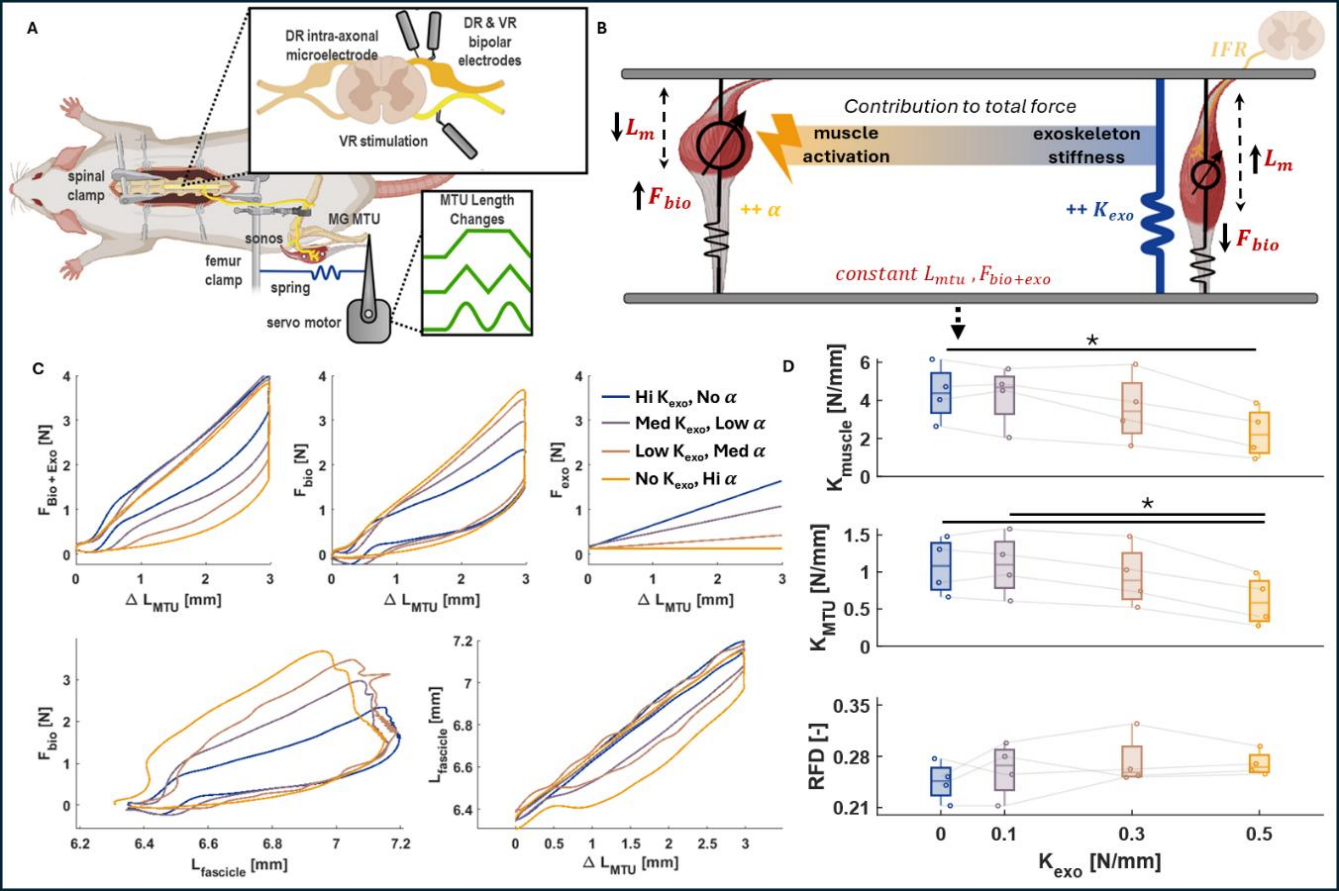
Experimental design A) animated representation of the experimental set up. A laminectomy is performed to expose the spinal cord, and bipolar electrodes are placed on isolated dorsal and ventral root fibers of interest. An intra-axonal microelectrode is inserted in the dorsal root to read afferent spikes. The femur is clamped, and the MG MTU is attached to a servo motor in parallel with exoskeleton springs. Muscle length changes are measured using sonomicrometry crystals, the muscle is stimulated through the ventral root. B) A figurative representation of the changes in muscle dynamics as we tradeoff between biological muscle and exoskeleton contributions to the total force. C) Muscle and MTU workloops from a representative rat. While the $F_{\text{bio+exo}}$ vs $L_{\text{mtu}}$ remains constant across all conditions, the tradeoff between muscle contribution and exoskeleton contribution is seen in the $F_{\text{bio}}$ vs $L_{mtu}$ (i.e. MTU stiffness) and $F_{\text{exo}}$ vs $L_{\text{lmtu}}$ (i.e. exoskeleton stiffness) curves. F_bio_ vs ${\text{L}}_{\text{fascicle}}$ represents the muscle stiffness, and ${\text{L}}_{\text{fascicle}}$ vs ${\text{L}}_{\text{mtu}}$ represents the relative fascicle displacement (RFD). D) Summary data from 4 rat models (shown in faded lines).

**Figure 3.**
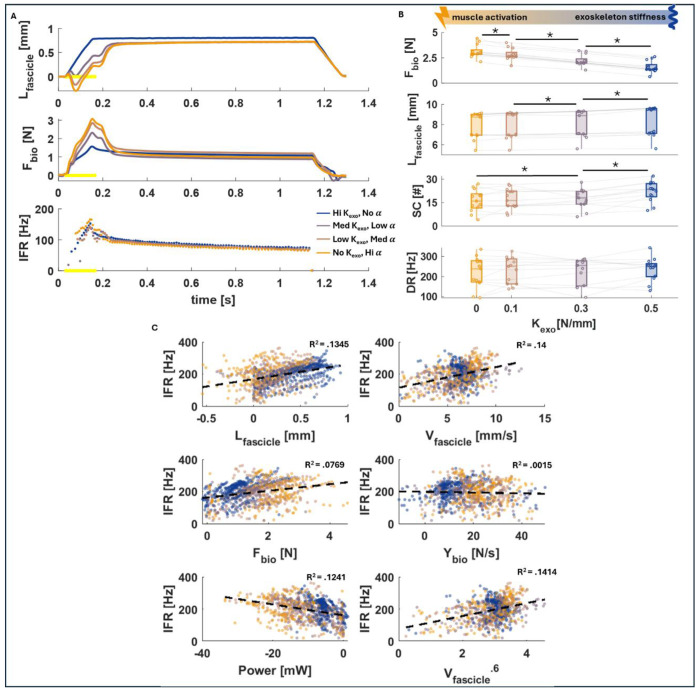
A) Time series fascicle length and force data, along with IFR, from a representative afferent cell. The total F_bio+exo_ is only held constant across all conditions during the period indicated by the yellow stimulation dots. The dots in the IFR occur at the time stamps in which a spike was recorded. B) Summarized data across all 13 cells (each represented by the faded lines). Significant changes are represented by a star and line between the two values. C) Correlation fits between the firing and various muscle properties across all cells. Correlations were fit to the controlled portions of the ramps and ignored the initial burst during the trials in which they occurred.

To our knowledge, this is the first in-situ investigation of the effects of exoskeleton assistance on spindle feedback in an animal model during active contractions. Our benchtop in situ setup effectively reproduced in vivo muscle-level dynamics observed from ultrasound and electromyography studies in humans using ankle exoskeletons (Fig. 1A) (8, 34). The unique mechanical context offered by parallel forces from an exoskeleton effectively decoupled kinetic and kinematic factors within the MTU and enabled the opportunity to clearly distinguish which biomechanical factors drive spindle primary afferent firing (Fig. 2B). As expected, trading-off increasing exoskeleton stiffness with decreasing muscle activation maintained a constant total exo+biological force output and decreases in muscle-tendon unit (MTU) force were accompanied by increases in muscle fascicle length (Fig 3A, 3B).

**Figure 4.**
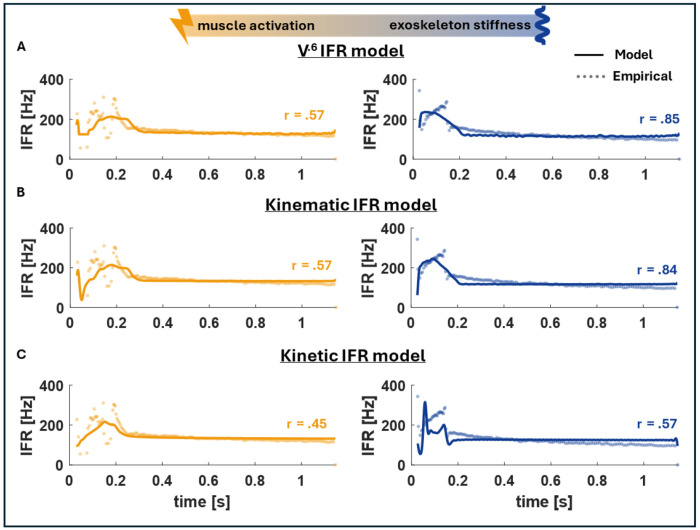
The time-series firing from a representative cell within the same rat model, overlaid over three firing models described in previous literature. A) The *V*^0.6^ model is represented by the equation $IFR_{v^{\cdot 6}}=\left(V^{0.6}+b_{v}\right)k_{v}$ (30). B) The Kinematic IFR model is represented by the equation $IFR_{L}=\left(\Delta L_{m}+b_{L}\right)k_{L}+\left(V_{m}+b_{V}\right)k_{V}$ (26). C) The Kinetic IFR model is represented by the equation $IFR_{F}=\left(F_{c}+b_{F}\right)k_{F}+\left(Y_{c}+b_{Y}\right)k_{Y}$ (26).

Interestingly, we observed no significant change in primary afferent IFR with exoskeleton assistance and there was no clear alignment between spindle firing and either the muscle fascicle kinetics or kinematics over the period of muscle-tendon unit stretch (Fig. 3B). A potential explanation for the lack of significant change in the dynamic response of the primary afferent firing is that the muscle spindle fibers may correlate with a more complex or nonlinear set of variables than the ones presented here (26, 38, 39). There have been numerous attempts to accurately represent the spindle firing and the dynamics of the intrafusal muscle fibers which the primary afferent endings are wrapped around (15). Classical approaches involve investigating different MTU and fascicle dynamics and their correlation with muscle spindle firing, but our results suggest that none of these proposed models can accurately account for the firing dynamics we measured (30) (Fig. 4A). More recently, Blum et al. showed that kinetic models outperform kinematic models in predicting IFR generated during passive stretches in-situ. Neither the kinetic nor kinematic models, however, predicted spindle firing in our data across exoskeleton conditions (Fig. 4B, 4C) (26).

One approach that may shed some light on the results in this experiment is via non-linear or viscoelastic models of the intrafusal and extrafusal muscle fibers to predict primary and secondary afferent firing (40–42). Other studies have pointed toward the potential that spindles encode more abstract representations of biomechanical state during locomotion. For example, Kwak et al. suggested that the fascicle dynamics sensed by the muscle spindles could reflect the whole-body walking economy (28). Furthermore, Monjo and Allen suggested more recently that afferent signals from muscle spindles may play a significant role in effort perception, suggesting that the brain receives information about the strength of fusimotor commands through an increase in afferent firing rate driven by gamma drives (43).

Our findings lend some support to the idea that sensory adaptation must occur from other mechanisms driven by descending commands, such as modulating gamma drive. Previous work has demonstrated significant sensorimotor adaptation to exoskeleton assistance (44–46), but few studies have attempted to identify the underlying neural mechanisms. For example, Kao et al. (44), demonstrated a 36% reduction in soleus EMG in humans walking with a powered ankle exoskeleton and in a follow up study (45) showed that soleus H-reflex modulation remains unaltered with powered exoskeleton assistance in unimpaired human users. This indicates that increased presynaptic inhibition of Ia afferents is likely not the mechanism by which soleus EMG is reduced with exoskeleton assistance. Although we found that changes in neuromechanical context due to exoskeleton assistive force had little effect on primary afferent feedback, a limitation of our study is that gamma drive is inhibited in these preparations. Therefore, the afferent feedback measured from the spindle fibers will not be the same as when the spindles are tuned via gamma activation. It is possible that the altered body-environment dynamics introduced by exoskeletons impact gamma drive in human users, and this could act to modify the output from spindle feedback. In addition, changes in motor output resulting from donning exoskeletons may result from modulation in higher-level cortical control that elicits a more direct attenuation of supra-spinal drive, especially in the early stages of learning the novel task (46).

Intrafusal muscle fibers have contractile dynamics independent of the extrafusal fibers they are embedded within. Muscle spindle firing, in turn, results from mechanotransduction of the muscle spindle group primary afferents in the nerve endings wrapped around these intrafusal muscle fibers. Understanding the interaction between these fiber types may, therefore, be necessary to explain the changes in muscle spindle firing due to an added exoskeleton in parallel to the MTU influencing extrafusal muscle fiber dynamics. The simple muscle model we developed here showed that the muscle kinetics and kinematics are decoupled due to added parallel stiffness to the MTU but cannot predict the resulting dynamics experienced by the intrafusal fibers that would explain the experimental results we found. Our recent biophysical muscle spindle model is a potential candidate for this (38, 47). The model simulates extrafusal muscle length changes based on the experimentally measured motor forces and applies this length to the two types of intrafusal fibers, bag 1 and chain, under the assumption that intrafusal fibers act in parallel with the extrafusal fiber. All muscle forces are modelled based on cross-bridge dynamics, with each fiber type having different properties of crossbridge dynamics. Each muscle fiber also receives its own independent motor drive—alpha drive to the extrafusal muscle, gamma static to the chain fiber, and gamma dynamic to the bag 1 fiber. The model also applies a normalized and scaled value of alpha activation that was used in the experiment and kept the gamma motor activation at a minimal level to simulate alpha activation below the gamma threshold. This model may, thereby, allow us to more accurately predict muscle spindle firing by simulating the dynamics that the intrafusal muscle fibers experience directly.

This study provides initial insight along a path to mechanistic understanding of how wearable robotic systems impact human sensorimotor integration during unsteady dynamic movements that require sensory feedback control. Here, we successfully replicated muscle-level dynamics observed in human ankle exoskeleton studies using a benchtop in-situ rat preparation by placing an elastic exoskeleton in parallel with the exposed medial gastrocnemius muscle tendon unit. Trading-off increasing exoskeleton stiffness with decreasing muscle stiffness drastically altered muscle force and length dynamics but did not significantly alter muscle spindle Instantaneous Firing Rate (IFR). This finding is critical for informing future muscle spindle feedback models and suggests that more complex biophysical models of intrafusal fiber dynamics must be developed. Furthermore, these findings imply that the nervous system’s adaptation to exoskeleton assistance may rely on higher-order modulation, such as changes in gamma drive or supraspinal control, rather than direct alterations in peripheral spindle sensitivity. Next experiments could employ decerebrate animal preparations to enable the study of exoskeleton interaction with in-vivo systems with intact spinal and peripheral circuitry but without higher cortical interference. This could provide a powerful framework to isolate impact of exoskeletons on reflexive and gamma-mediated control mechanisms. Complementary experiments using microneurography in humans could help extend these insights to understand if and how exoskeletons could augment human stability or agility during sensorimotor control tasks requiring rapid response to unsteady conditions and whether humans can adapt to robotic assistance over longer timescales to become super-human movers.

## Materials and Methods

We designed a muscle contraction paradigm where the muscle-tendon length was clamped and force was matched across various exoskeleton stiffness conditions, to resemble joint-level dynamics in standing balance. The length was clamped via a servo motor in series with the MTU, and the force was matched by trading off between the muscle force (controlled via VR stimulation) and the exoskeleton stiffness. We recorded the increase in muscle fascicle length alongside a decrease in the force with increased exoskeleton stiffness, while recording the primary afferent firing.

### Simple Muscle Model

Our muscle model builds on the model developed by Blum et al., in which a muscle and muscle spindle are represented in parallel with a tendon in series with both (38). Here, we split the muscle into its contractile and non-contractile elements with a tendon in series, and a passive exoskeleton spring in parallel to the whole MTU (Fig. 1A). Length changes ($L_{\text{in}}$) were applied to the model while modulating active stiffness of the muscle to maintain a constant total force output ($F_{\text{total}}$), including the muscle force (${\text{F}}_{\text{bio}}$) and exoskeleton force (${\text{F}}_{\text{exo}}$). Parallel passive exoskeletons of varying stiffness were added to the MTU to determine its effect on the predicted spindle IFR. The biological muscle force was split into contractile $\left(F_{c}\right)$ and noncontractile $\left(F_{NC}\right)$ components. $F_{NC}$ was determined according to $(38):F_{NC}=k_{\text{lin}}\left(\Delta L_{m}\right)+Ae^{k_{exp}\left(\Delta L_{m}\right)}$, where $\Delta L_{m}$ is the muscle length change, and $k_{\text{lin}},k_{exp}$, and A are optimized fit constants. $F_{c}$ was determined by subtracting $F_{NC}$ from $F_{\text{bio}}\text{:}F_{\text{bio}}=F_{C}+F_{NC}.F_{c}$ was then used to predict IFR in a kinetic based IFR model: $IFR_{F}=\left(F_{c}+\right.\left.b_{F}\right)k_{F}+\left(Y_{c}+b_{Y}\right)k_{Y}+C$, where $Y_{C}$ is the Yank, or the force time derivative of $F_{C}$, and $b_{F},k_{F},b_{Y},k_{Y},C$ are optimized fit constants. Similarly, the muscle length change and velocity ($V_{m}$) were used to predict IFR in a kinematic based IFR model $IFR_{L}=\left(\Delta L_{m}+b_{L}\right)k_{L}+\left(V_{m}+b_{V}\right)k_{V}+C$.

To understand the expected muscle dynamics and decoupling of the muscle length and force changes with added exoskeleton stiffness, it helps to visualize the system in terms of muscle stiffness (muscle force divided by muscle length). With an added exoskeleton stiffness ($k_{\text{exo}}$) in parallel to the MTU, the total force was split between the exoskeleton and the MTU, resulting in the following stiffness characteristics.


1) $$K_{\mathrm{MTU}+\mathrm{exo}}=\frac{F_{\mathrm{mtu}}+F_{\mathrm{exo}}}{L_{\mathrm{mu}}}$$

2) $$K_{\text{MTU}}=\frac{K_{m}K_{T}}{K_{m}+K_{T}}$$

3) $$K_{\mathrm{MTU}+\text{exo}}=\frac{K_{m(w/\text{exo})}K_{T}}{K_{m(w/\text{exo})}+K_{T}}+K_{\text{exo}}$$

4) $$K_{m(w/\mathrm{exo})}=\frac{K_{T}\left(K_{\mathrm{mtu}+exo}-K_{exo}\right)}{K_{T}+K_{exo}+K_{mtu}+exo}$$

With $K_{T}$ (tendon stiffness) and $K_{\text{mtu+exo}}$ as constants, equation (4) shows that as the exoskeleton stiffness increases, the muscle stiffness ($K_{m}=\frac{F_{\text{bio}}}{\Delta L_{m}}$) decreases, and vice versa (Fig. 1D). As a result, IFR decreased as the parallel exoskeleton stiffness increased when determined as a function of muscle kinetics but increased when determined as a function of muscle kinematics (Fig. 1C). These contrasting predictions of kinematic vs. kinetic models for spindle output IFR under ramp muscle loading helped inform the in-situ investigation in a rat model detailed in this study.

### Animal Care

All procedures and experiments were approved by the Georgia Institute of Technology Institutional Animal Care and Use Committee (protocol number A100142). The surgical methods performed were similar to those carried out in Abbott et al. (27). Briefly, Adult female Wistar rats (250–300 g; Charles River Laboratories, Wilmington, MA, USA) were studied in terminal experiments in which they were deeply anaesthetized using isoflurane initially in an induction chamber (5% in 100% O2) and for the remainder of the experiment via a tracheal cannula (1.5–2.5% in 100% O2) while vital signs were continuously monitored. Anesthesia level and vital signs were maintained by adjusting isoflurane concentration, radiant and water-pad heat sources, and by scheduled subcutaneous injection of lactate ringer solution (1 ml/h subcutaneous).

### Data collection

IFR was measured via a glass microelectrode inserted intracellularly into the L5 dorsal root while it was suspended on bipolar hook electrodes to record the activity of single cells. Afferent firing was recorded while imparting a predetermined length change in the form of ramps (3 mm amplitude, 0.125 s rise and fall time, 1 s hold, 24 mm/s rising speed), sinusoids (4 hz, 3 mm peak-peak), and triangles (4 hz, 3 mm peak-peak) using a servo motor (Aurora Scientific (Ontario, Canada)) attached to the Achilles tendon. The femur was clamped in place, and the Medial Gastrocnemius (MG) was isolated from the other plantar flexors, and activation of the isolated muscle is modulated with electrical stimulation imparted through the ventral root efferents. 100 Hz stimulation was imparted at amplitudes dependent on the total MTU and exoskeleton force required during the up ramp of the ramp and hold stretches. Muscle fascicle length was measured using sonomicrometry crystals embedded in the MG muscle belly (Sonometrics (Ontario, Canada)), taking care to avoid the aponeurosis. Various springs were attached (ranging from 0.1 to 0.5 N/mm) in parallel to the medial gastrocnemius MTU (to the servo motor distally and the femur clamp proximally) to simulate a passive exoskeleton (Fig. 2A). The resulting length clamped, force-matched eccentric contraction across various exoskeleton stiffness conditions, as depicted in Figure 1B, resembled standing balance paradigms with ramp platform perturbations with and without an exoskeleton (8). All data were collected with a Cambridge Electronic Design Power 1401 and using Spike2 software and analyzed in MATLAB.

The springs used to represent a passive exoskeleton were attached via a custom tensioning mechanism by which the preset tension in the springs could be modified to have a consistent initial exoskeleton force acting across all spring conditions. The extension springs (McMaster-Carr) had hook ends and were all the same initial length, so that once the tensioning mechanism was adjusted for the specific rat model’s MTU length, all springs could be easily swapped throughout the experiment to test across all experimental conditions.

### Data Processing and Analysis

Measurements of motor position, motor force and fascicle displacement lowpass filtered with a fourth-order Butterworth filter with a 50 Hz cutoff. They were then differentiated with respect to time to get the yank $\left(\frac{dF}{dt}\right)$, the velocity $\left(\frac{dL}{dt}\right)$, and the acceleration $\left(\frac{d^{2}F}{dt^{2}}\right)$. Fascicle power was calculated as the fascicle force multiplied by the fascicle velocity. The total MTU and Exoskeleton system displacement and force were measured by the servo motor’s displacement and measured force. The exoskeleton force was calculated as the passive spring stiffness multiplied by the displacement of the spring, which was equivalent to the displacement of the total MTU and exoskeleton system. The tensioning mechanism was adjusted for each animal to ensure that the springs always start at resting length, and the initial exoskeleton force is therefore zero. The biological MTU force was then calculated as the total force minus the exoskeleton force. This was further validated experimentally by comparing passive stretches of the total MTU and exoskeleton system across all spring conditions (no spring, 0.1 N-mm, 0.3 N-mm, 0.5 N-mm). This was done by subtracting the passive, no spring stretch condition from the other spring conditions and then fitting a linear function to the resulting force length curve (i.e. the stiffness of the spring in the exoskeleton’s force contribution).

To validate the effects of the parallel exoskeleton, the length change of the fascicle relative to the MTU, the stiffness of the MTU, and the stiffness of the fascicle were computed for each exoskeleton condition type. Stiffness (N/mm) was computed by fitting a linear function to force/length curves during the up-ramp of the ramp-and-hold stretches. Stiffness (in N/mm) was calculated by computing the slope of the tangent line at these force thresholds. The relative fascicle displacement (RFD) was determined in the same manner, estimating the slope $dL_{\text{fas}}/dL_{\text{MTU}}$ (Fig. 2D).

Spike count during the up-ramp, dynamic responses, and static responses were extracted from firing rates of the ramp- and-hold stretches (Fig. 3B). The dynamic response was taken as instantaneous firing rate at the peak MTU force production, when it first reaches its max stretch. The static response was taken as the firing rate approximately 0.5 s into the hold phase, and the dynamic index is the difference between the dynamic and static.

Statistical comparisons of firing metrics and muscle dynamics with exoskeleton stiffness for each length change type were conducted using repeated measures ANOVA in MATLAB. Data was collected from 24 unique primary afferent cells across 8 animals and reduced to 13 cells across 4 animals after filtering based on the exclusion criteria mentioned above, along with the availability of all exoskeleton conditions for that cell. Linear regressions were computed in MATLAB and ignored the initial burst in the firing. The regressions only consider the rising edge of the ramp stretch, because that is the period during which the contraction was controlled using stimulation to match the total length and force change of the MTU + Exo system. Reported values are the mean ± SEM unless stated otherwise. Data were excluded from analysis if the sensory afferent action potentials could not be discriminated and/or if muscle dynamics did not reflect those expected from standing balance trials with an exoskeleton (i.e. decreased muscle force production and increased muscle stretch with increased parallel exoskeleton stiffness).
